# Transgene-free genome editing in marine algae by bacterial conjugation – comparison with biolistic CRISPR/Cas9 transformation

**DOI:** 10.1038/s41598-018-32342-0

**Published:** 2018-09-26

**Authors:** A. K. Sharma, M. Nymark, T. Sparstad, A. M. Bones, P. Winge

**Affiliations:** Cell, Molecular biology and Genomics Group, Department of Biology, Norwegian University of Science and Technology, N-7491 Trondheim, Norway

## Abstract

The CRISPR/Cas9 technology has opened the possibility for targeted genome editing in various organisms including diatom model organisms. One standard method for delivery of vectors to diatom cells is by biolistic particle bombardment. Recently delivery by conjugation was added to the tool-box. An important difference between these methods is that biolistic transformation results in transgene integration of vector DNA into the algae genome, whereas conjugative transformation allows the vector to be maintained as an episome in the recipient cells. In this study, we have used both transformation methods to deliver the CRISPR/Cas9 system to the marine diatom *Phaeodactylum tricornutum* aiming to induce mutations in a common target gene. This allowed us to compare the two CRISPR/Cas9 delivery systems with regard to mutation efficiency, and to assess potential problems connected to constitutive expression of Cas9. We found that the percentage of CRISPR-induced targeted biallelic mutations are similar for both methods, but an extended growth period might be needed to induce biallelic mutations when the CRISPR/Cas9 system is episomal. Independent of the CRISPR/Cas9 vector system, constitutive expression of Cas9 can cause re-editing of mutant lines with small indels. Complications associated with the biolistic transformation system like the permanent and random integration of foreign DNA into the host genome and unstable mutant lines caused by constitutive expression of Cas9 can be avoided using the episomal CRISPR/Cas9 system. The episomal vector can be eliminated from the diatom cells by removal of selection pressure, resulting in transient Cas9 expression and non-transgenic mutant lines. Depending on legislation, such lines might be considered as non-GMOs.

## Introduction

Diatoms are a species-rich group of unicellular, photosynthetic eukaryotes found worldwide in fresh waters and oceans, with enormous ecological significance^1,2^. This group of photosynthetic microalgae also has a multitude of potential applications within bio-, nano- and environmental technologies. Diatoms can be used as a source of lipids for biofuel production, as an ingredient in fish feed, as a nitrogen-fixing biofertilizer, in industrial waste detoxification, and in the synthesis of biomaterial and computer chips^3–5^. Until the last couple of years, development of diatom strains for industrial use have been hampered by limited tools for genetic manipulation. New tools such as TALEN, Meganuclease, and CRISPR/Cas9-based methods have been developed and recently been adopted for diatoms^6–8^. The availability of efficient techniques for manipulation of diatom genomes enable a reverse genetics approach for studies of the complex biology of diatoms, and strain optimization to lower the production cost of relevant commercial products.

In diatoms, the successful use of the CRISPR/Cas9 system to introduce targeted genome modifications has been based on biolistic-mediated delivery of CRISPR-Cas9 plasmids^7,8^. Recently it was demonstrated that Cas9 gene editing could also be performed by delivering the CRISPR/Cas9 plasmid by bacterial conjugation^9^. Biolistic DNA delivery has been the most common technique for transformation of diatoms. This system requires high plasmid concentrations, results in relatively low transformation rates, cell damage after microprojectile bombardment and stable integration of functional parts of the plasmid DNA into the host genome which may affect random genetic elements^10^. Introduction of the components of the CRISPR/Cas9 system in this way results in transgenic diatom lines with high expression of Cas9 and sgRNA. The mutation efficiency at the desired target site is high using the biolistic approach^7,8^. Stable expression of Cas9 has however been reported to increase the probability of off-target effects^11^. Considering the above-described drawbacks of biolistic transformation, we have constructed (in parallel to work by Slattery and co-workers^9^) a system for performing conjugative CRISPR/Cas9-mediated gene editing. Transformation of diatoms by bacterial conjugation is highly efficient, requires no expensive equipment, avoid incorporation of “foreign” DNA into the diatom genome, and provides the opportunity for transient expression of Cas9 and the creation of transgene-free Cas9-edited lines.

We present here a direct comparison between the efficiency of Cas9-mediated mutagenesis using bacterial conjugation compared to using biolistic transformation methods for the delivery of CRISPR-Cas9 plasmids to diatom cells. The same gene encoding a transcription factor belonging to the Myb family^12^, was targeted for editing using the two different approaches for delivery of the CRISPR components. Mutation efficiency, advantages and disadvantages associated to the choice of CRISPR/Cas9 plasmid delivery system are discussed. We also show that episomes are stably maintained in *P*. *tricornutum* as long as they grown under antibiotic positive selection.

## Results and Discussion

**Figure 1:**
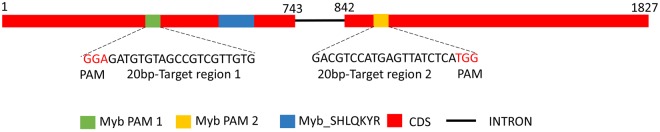
Schematic illustration of the Myb target gene. Exons are indicated by solid filled blocks and target region Myb PAM1 (green block), and Myb PAM2 (orange block) lies within the coding region of the gene. The single Pt MYB1R domain (SHLQKYR) is indicated by a solid blue block. The intron is indicated by a thin black line. The target sequence for Myb PAM1 and PAM2 are presented below. Numbers indicate nucleotide position within the gene sequence.

To enable the expression of the components necessary for CRISPR/Cas9-mediated mutagenesis from a nuclear episomal vector, the Cas9-gRNA cassette from the pKS diaCas9_sgRNA vector^7^ was inserted into a modified version of the pPtPuc3 vector (pPtPuc3m)^13^. The origin of transfer (*oriT*) in the pPtPuc3m backbone allows the delivery of the plasmid to *P*. *tricornutum* cells by bacterial conjugation. The vector map for the pPtPuc3m diaCas9_sgRNA plasmid is presented in Supplementary Fig. 1. The mutation efficiency of Cas9-mediated gene disruption in cells containing the episomal CRISPR/Cas9 vector (pPtPuc3m diaCas9_sgRNA) was compared to the efficiency achieved in cells where the CRISPR/Cas9 vector (pKS diaCas9_sgRNA) was integrated into the *P*. *tricornutum* genome using biolistic transformation. A gene encoding a Myb transcription factor (Pt_Myb1R_SHAQKYF5)^12^ was selected for targeted gene editing using the two different CRISPR/Cas9 vectors and vector delivery techniques. Two sgRNAs targeting different regions (Myb PAM1 and PAM2; Fig. 1) of the Myb coding sequence were designed, and vectors expressing both sgRNAs were delivered by conjugation and biolistic transformation.

### Delayed appearance of CRISPR mutants in colonies expressing Cas9 from the episomal vector

**Figure 2:**
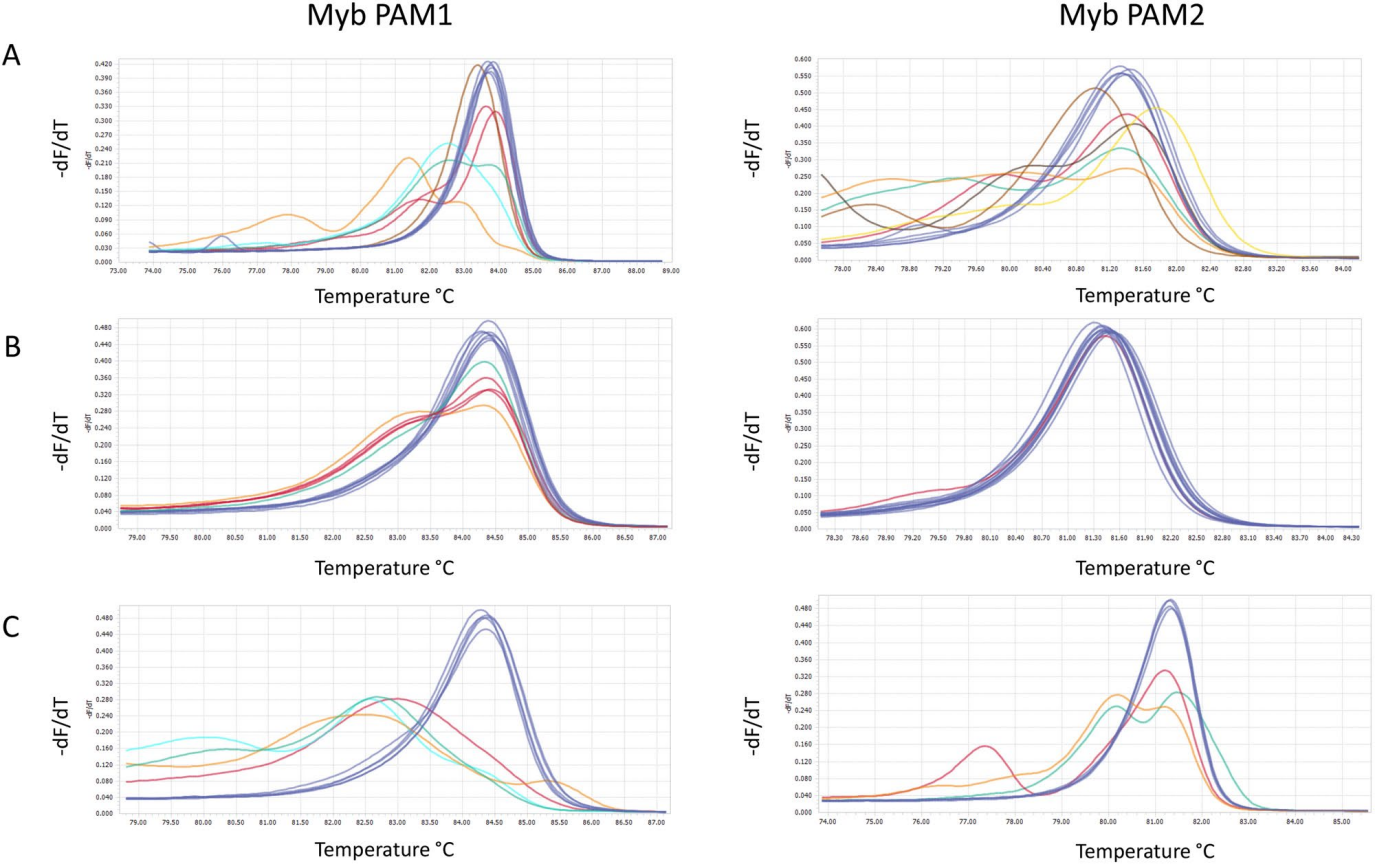
Screening of transformants for Cas9-derived mutations in the Myb PAM1 and Myb PAM2 target regions. High resolution melting (HRM) analyses data presented as normalized melting peaks generated from PCR products from (**A**) primary colonies resulting from transformation with pKS diaCas9_sgRNA using biolistic bombardment (one month after transformation; n = 12) (**B**) primary colonies resulting from transformation with pPtPuc3m diaCas9_sgRNA using bacterial conjugation (one month after transformation; n = 12); (**C**) re-plated colonies resulting from transformation with pPtPuc3m diaCas9_sgRNA (three months after transformation; n = 12). WT profiles are represented by dark blue lines. Differently colored melting peaks indicate a melting behavior unlike that of the WT PCR product and implies the presence of indels in the Myb PAM1 and PAM2 target regions. Normalized melting peaks were generated by LightCycler® 96 Application Software Version 1.1.

**Figure 3:**
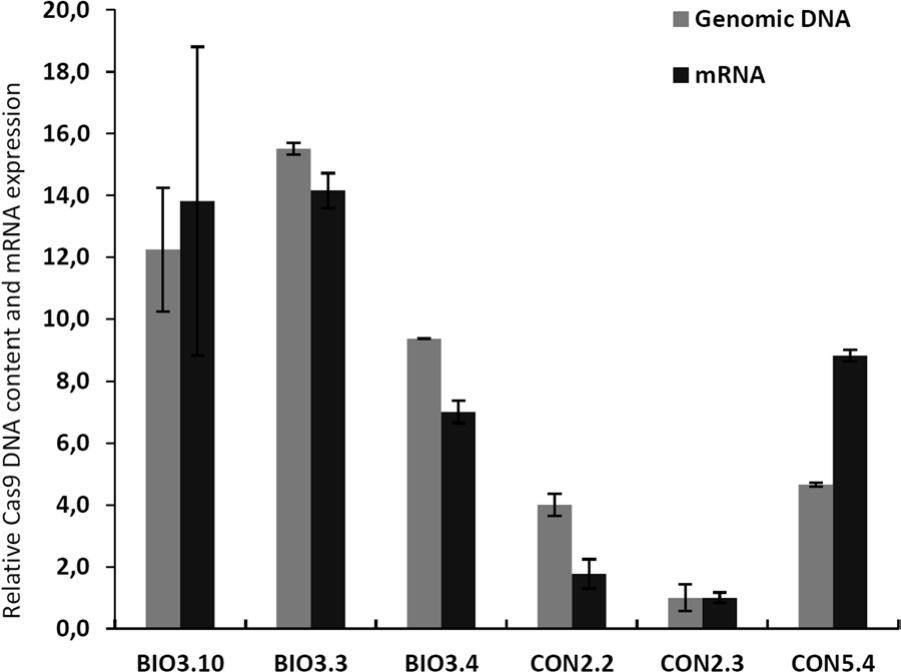
Comparison of Cas9 mRNA expression levels versus genomic/episome Cas9 DNA content in biallelic mutant clones generated using biolistic bombardment or bacterial conjugation methods for CRISPR/Cas9 plasmid delivery. The grey bars represent relative Cas9 gene expression values and the black bars relative level of genomic/episome Cas9 DNA. Expression of Cas9 is the average of three biological replicates for each line and is normalized to the mutant line displaying the lowest Cas9 expression level (CON2.3). The relative amount of Cas9 DNA is normalized to CON2.3 the cell lines showing lowest Cas9 DNA content. Error bars represent the 95 percent confidence intervals. The abbreviations used are BIO: Cas9 editing lines resulting from biolistic CRISPR/Cas9 plasmid delivery; CON: Cas9 editing lines resulting from delivery of CRISPR/Cas9 plasmid by bacterial conjugation.

Delivery of plasmid DNA to diatom cells by bacterial conjugation results in increased transformation rates and reduced incubation time after transformation compared to the biolistic technique^13,14^. In our study, transformed colonies appeared 2 weeks after transformation by bacterial conjugation and 3–6 weeks after biolistic transformation, which is in agreement with previous reports^7,13^. The transformation efficiency was approx. 20–100 times higher when employing conjugation compared to biolistic delivery (Table 1). Screening of colonies for cells containing Cas9-derived mutations were achieved by a combination of high resolution melting (HRM) analyses and Sanger sequencing of the two target areas in the Myb gene. The first round of screening of primary colonies were performed one month after transformation with the two different CRISPR/Cas9 plasmids. Targeted gene modifications were detected in 33–50% of the primary colonies (n = 12) where the pKS diaCas9_sgRNA plasmid had been delivered using the biolistic system (Fig. 2A, Table 2). In contrast, the fraction of edited cells indicated by HRM analysis of one-month-old primary colonies (n = 12) resulting from conjugative delivery of the pPtPuc3m diaCas9_sgRNA plasmid was lower (Fig. 2B) and could not be confirmed by direct sequencing of the target region. Cells from these seemingly non-mutated conjugative transformants were transferred onto new selection plates once per month and re-analyzed three months after transformation. The results from this additional screening revealed that a prolonged growth period of cells expressing the Cas9 and guide RNA from the episomal vector, produced Cas9-derived mutations in 25–33% of cells originating from colonies derived from the primary screen (Fig. 2C, Table 2). A possible explanation for the delayed appearance of mutants in these transformants, could be a reduced Cas9 gene expression as a consequence of lower episomal CRISPR-Cas9 plasmid copy number (1–2 copies;^13^) compared to the copy number of integrated CRISPR-Cas9 plasmid resulting from biolistic transformation (1–10 copies;^14^). However, transcriptional analysis of mutant lines generated with both vectors and transformation systems, indicate that the Cas9 gene expression is not consistently lower from the episomal CRISPR-Cas9 plasmid compared to lines containing integrated plasmids (Fig. 3). To compare the Cas9 mRNA expression levels with the amount of genomic Cas9, either as integrated in the genome or as part of the episome, a semi quantitative qPCR was performed on genomic DNA from the transformed lines. The result shows a good correlation between mRNA expression and genomic Cas9 levels for the individual samples with in average more than 3 times higher Cas9 DNA levels in the three lines generated using the biolistic method compared to lines containing episomes (Fig. 3). Another plausible explanation for the delayed detection of mutant cells after conjugative transformation, could be a high rate of cell division with a short or no lag phase after transformation. If the transformed cells manage to go through several cell divisions before the Cas9 nuclease induce a mutagenic event, the primary colonies might contain a low fraction of cells with mutations compared to WT cells, in this case below or at the detection limit in our screening system. The cell damage inflicted by the impact of tungsten particles with a size that is as much as 1/3 of the width of the *P*. *tricornutum* cells, is likely to cause a stop in cell division until the damage has been repaired. The recovery time needed after transformation by biolistic bombardment is probably the reason for the prolonged growth period before transformed colonies can be observed, compared to the incubation time necessary for colonies to appear after transformation by bacterial conjugation. The halted or slow cell division after biolistic transformation might also increase the chances of Cas9-mutagenesis to take place before or within the first couple of cell divisions, thereby increasing the fraction of edited alleles within the primary colonies. In contrast, no cell damage is expected after transformation by conjugation, facilitating multiple cell divisions to take place before Cas9 induced mutations have been produced, resulting in a high fraction of WT cells in the primary colonies. This is also reflected as an increased variation in the types of indels (Supplementary Fig. 2).

**Table 1:** Comparison of features connected to use of the biolistic bombardment method and bacterial conjugation system for CRISPR/Cas9 plasmid delivery to diatom cells.

	Biolistic	Conjugation
Transformation rate	5–25 transformants/10^8^ cells	500–1000 transformants/10^8^ cells
Cell damage	Yes	No
Plasmid used per transformation	2.5 µg of each plasmid	200 ng
Incubation time	3–6 weeks	2 weeks
Mutation efficiency	Higher	Lower
Plasmid integration in genomic DNA	Yes	No
Possibility for removal of CRISPR components	No	Yes
Possibility for off-target effects	Yes	Not after elimination of CRISPR/Cas9 episome
Possibility for re-editing	Yes	Not after elimination of CRISPR/Cas9 episome
Mutants regulated as GMO	Yes (transgenic)	Not always if CRISPR episome has been eliminated (non-transgenic)

**Table 2:** Mutation efficiency of the two different CRISPR/Cas9 vectors and vector delivery systems.

	Colonies in total	Screened colonies	Edited colonies	Percentage edited colonies
**Myb PAM 1**				
Biolistic	19	12	6	50%
Bacterial Conjugation	900 +	12	4	33%
**Myb PAM 2**				
Biolistic	15	12	4	33%
Bacterial Conjugation	500+	12	3	25%

The presented data is from one-month-old primary colonies resulting from the introduction of the pKS diaCas9_sgRNA vector using biolistic bombardment, and three-months-old (re-plated once per month) colonies transformed by delivery of the pPtPuc3m diaCas9_sgRNA vector by bacterial conjugation. Edited colonies were identified by HRM and targeted gene mutations were confirmed by sequencing.

### Mutation efficiency of the two different CRISPR/Cas9 vectors and vector delivery systems

To estimate the mutation efficiency achieved by using the two different CRISPR/Cas9 vectors and vector delivery systems, we compared the percentage of primary colonies containing cells with targeted mutations in one-month-old colonies transformed using the biolistic approach, to three-month-old re-plated colonies where the CRISPR-Cas9 plasmid had been introduced via conjugation. Our results show that for both Myb sgRNAs the highest number of Cas9-derived mutant colonies were found among the primary colonies that resulted from delivery of CRISPR-Cas9 plasmid by biolistic bombardment (Table 2). The mutation efficiency of 50% for Myb PAM-target site 1 and 33% for PAM-target site 2 is within the same range previously reported for this CRISPR/Cas9-system^7^. The extended growth period before detection of mutants, and the somewhat lower mutation efficiency (Myb PAM1: 33%; Myb PAM2: 25%) in cells expressing the Cas9 and sgRNA from an episomal vector was noticeable. The CRISPR/Cas9 biolistic bombardment system depends on co-delivery of two plasmids, one for expression of the *Sh ble* selection marker (pAF6), and one for expression of the Cas9 and sgRNA (pKS diaCas9_sgRNA). 30–40% of the colonies can be expected to contain only the pAF6 vector^14^, precluding Cas9-derived mutagenesis. Also, the physical force involved in the biolistic bombardment process can cause fragmentation of the pKS diaCas9_sgRNA plasmid resulting in incomplete Cas9 and/or sgRNA gene cassettes^15^. Delivery of the pPtPuc3m diaCas9_sgRNA plasmid by bacterial conjugation could potentially avoid these problems. The *Sh ble*, Cas9 and sgRNA genes are all located on the same episomal vector so that all components necessary for Cas9-mutagenesis should be present in all transformants. However, episome rescue of the original pPtPuc3 vector from *P*. *tricornutum* cells showed that only 50% of the episomes retained the original size, indicating that rearrangements of the diatom episome can occur during propagation in *E*. *coli* or during conjugative plasmid introduction^13^. However, pPtPuc3m diaCas9_sgRNA episomes rescued from the conjugation lines used in this experiment shows that they are intact and of correct size (Supplementary Fig. 5) and can therefore not explain the lower mutation efficiency in these lines. The episome instability may be related to its propagation in *E*. *coli* as the episome contain an origin of replication (Ori) derived from pUC19, which results in high plasmid copy number^16^. In combination with the large size of the episome ~ 12.8 Kb this may in some cases lead to instability and rearrangements. The integrity of the episome should therefore always be checked before it is used in conjugation experiments, see methods section (Transformation by bacterial conjugation). A solution for this may be to use episomes based on the pBR322 vector, such as pPtPBR11 (Addgene #80386)^17^. pBR322 has the same MB1 Ori as pUC19, but it also contains the Rop gene, which regulates plasmid copy number to lower levels. An alternative solution is to change a specific mutation found in the Ori of pUC19 derived vectors, including pPtPuc3 and pKS diaCas9_sgRNA, (a G → A in the RNA II locus), which may regulate plasmid copy number^16^. Differences in mutation efficiencies due to different transformations methods has also been reported in plants, where the mutation efficiency is significantly higher when introducing the Cas9 and sgRNA via *Agrobacterium*-mediated transformation^18–21^, than by biolistic transformation^15,22,23^.

To estimate the mutation frequency within a colony, indicated by the HRM analyses to contain cells with mutations, PCR products spanning the regions targeted for gene editing were TOPO-cloned, and 6–8 TOPO-clones were sequenced per PCR product/colony. The fraction of Cas9-edited alleles detected within each of the mutated colonies were found to be similarly high (in average >80% for both systems) and seemed to be independent of whether the CRISPR/Cas9 components had been incorporated into the diatom genome as a result of biolistic bombardment or expressed from the episomal CRISPR/Cas9 vector (Table 3). An overview of the different mutations generated by the two CRISPR/Cas9 gene editing and transformation systems are presented in Supplementary Fig. 2. Small indels causing frameshifts were the dominating type of mutations, but also small indels resulting in in-frame mutations and larger indels with a size of several hundred bp were detected. Slattery and coworkers^9^ detected conjugative Cas9-derived mutations in the urease gene in 10–60% of the primary colonies, but phenotypic screening for bi-allelic knockouts among subclones from these primary colonies revealed that only an average of ~10% of the subclones showed the expected phenotype. These results can be explained by either a high amount of in-frame mutations not affecting the urease enzyme activity, a high number of cells containing mono-allelic mutations, a low mutations frequency within the primary colonies or a combination of these events. A prolonged growth period before subcloning could have increased the mutations frequency of the gene of interest within each colony as seen in our experiment, but the different mutation efficiency seen for various sgRNAs and their target sites makes comparisons across experiments difficult.

**Table 3:** Percentage of Cas9-edited alleles in mutant colonies.

	Sequenced clones	Edited sequences	Percent of edited sequences
**Biolistic**			
Myb PAM 1 colony 1	6	5	83
Myb PAM 1 colony 3	8	7	87
Myb PAM 1 colony 4	7	6	85
Myb PAM 1 colony 5	7	5	71
Myb PAM 1 colony 8	7	6	85
Myb PAM 1 colony 10	7	7	100
**Bacterial conjugation**			
Myb PAM 1 colony 5	7	6	85
Myb PAM 1 colony 7	7	7	100
Myb PAM 1 colony 8	8	6	75
Myb PAM 1 colony 10	7	6	85
**Biolistic**			
Myb PAM 2 colony 2	7	5	71
Myb PAM 2 colony 4	8	6	75
Myb PAM 2 colony 9	8	8	100
Myb PAM 2 colony 10	8	7	87
**Bacterial conjugation**			
Myb PAM 2 colony 2	6	4	66
Myb PAM 2 colony 5	7	6	85
Myb PAM 2 colony 10	7	5	71

PCR products spanning the Myb PAM1 or Myb PAM2 target sites from biolistic transformation and bacterial conjugation were TOPO-cloned and sequenced to check for mutation at target sites.

### Consequences of constitutive expression of Cas9 over a prolonged period of time

**Figure 4:**
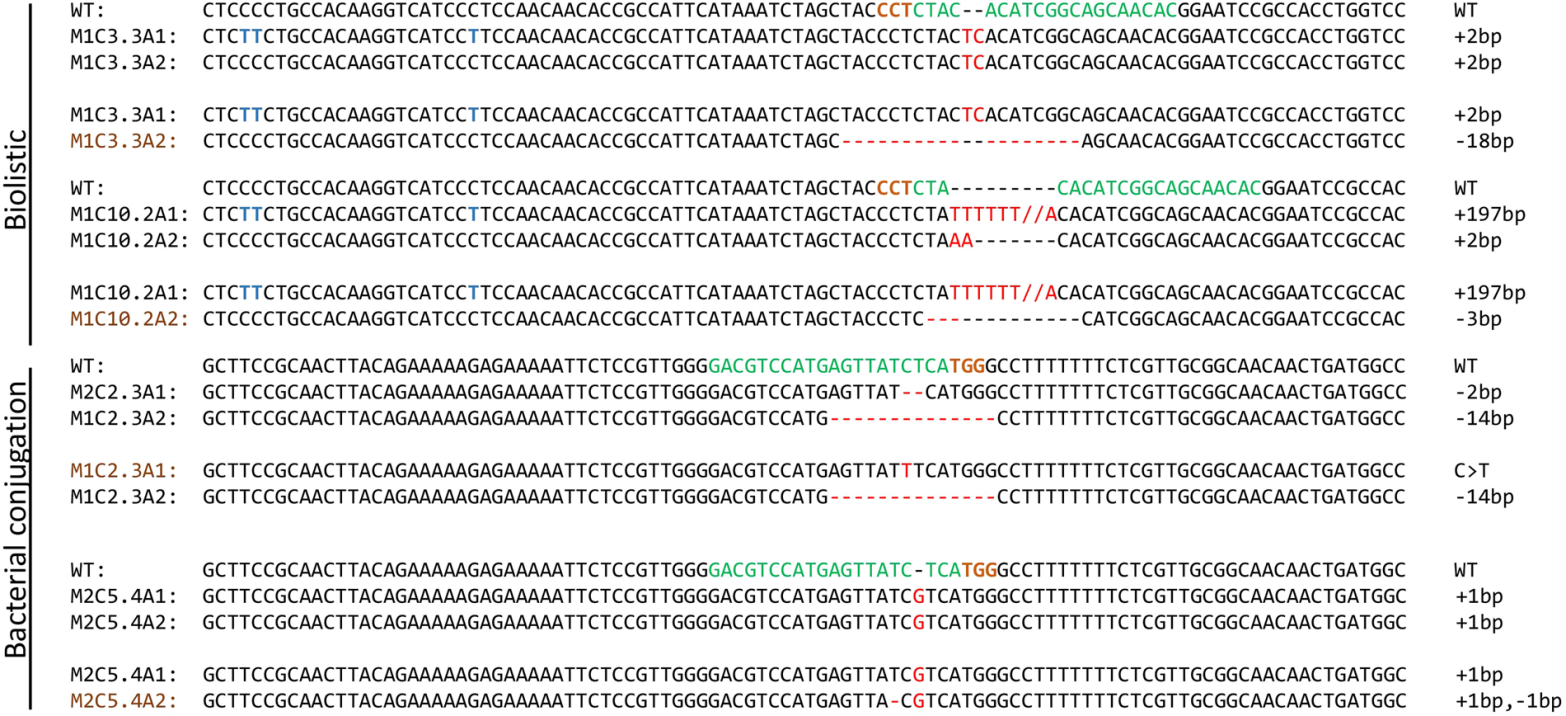
Re-editing of target regions caused by constitutively expressed Cas9 and sgRNA. Names of alleles in black font represent the first editing event, whereas names in brown font represent second editing. The target regions are indicated by bold green font with its respective PAM sequence in bold brown font. All indels are shown in bold red font. Polymorphisms present in the Myb gene (full overview in Supplementary Table 3) are indicated by blue bold font and were used to differentiate alleles for MYB1R_SHAQKYF5 gene.

The CRISPR/Cas9 system can tolerate up to 5 bp mismatches causing unintended double strand breaks and mutations at undesired regions^11,22,24,25^. Continuous and high expression of Cas9 and sgRNA increases the chances of off-target effects. In our study, cultures derived from subclones with confirmed biallelic out of frame mutations in the Myb gene were grown for a prolonged period (weeks to years). Re-sequencing revealed that in some cases continuous Cas9 expressing lines with small indels of 1–2 bp had been re-edited causing a change from an out of frame to an in-frame mutation in one of the alleles (Fig. 4). Re-editing was seen in both of the Myb target regions and took place even if the mismatches between sgRNA and target sequences were located in the seed sequence believed to be critical for sgRNA specificity^22,26,27^.

Re-editing incidents were independent of transformation system. In cases where re-editing of a gene restores or partially restores the WT phenotype, the re-edited cells might outcompete the knockout mutant cells and take over the culture within a few weeks of exponential growth. It is therefore essential that verification of knock out mutants known to possess small indels is performed before start-up of experiments aiming to characterize the phenotype of Cas9 expressing mutant lines.

### Non-transgenic Cas9 edited lines can easily be isolated

**Figure 5:**
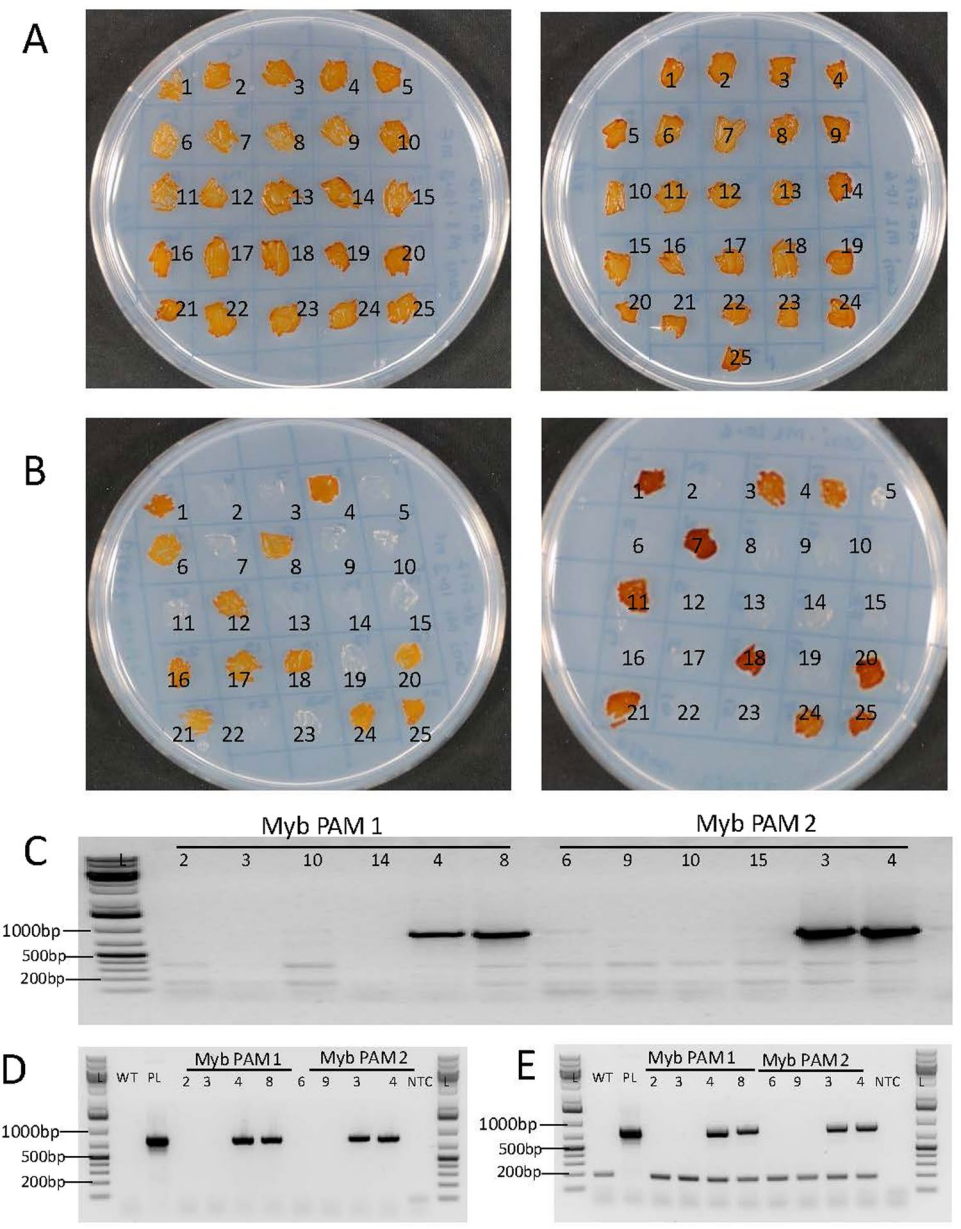
Identification of non-transgenic mutants. 25 colonies from Myb PAM 1 (left side) and Myb PAM2 (right side) patched on 50% seawater (SW) f/2 agar plate without zeocin (**A**) and with zeocin (**B**). All patched colonies survived on non-selection plates whereas only colonies still containing pPtPuc3m-Cas9_sgRNA survived on selection plates. Colonies lacking the plasmid appeared white and dead after ten days on selection plates. Presented pictures were taken seven days after patching. Loss of pPtPuc3m-Cas9_sgRNA episome in colonies not able to survive on selection plates was verified by the inability to amplify a Cas9 DNA fragment (817 bp) from these colonies (**C**). Cas9 PCR products generated from colonies still able to grow on selection plates were included as positive controls. Ten months after initial transformation episome free colonies and colonies containing episomes were reanalyzed for presence of Cas9 DNA (817 bp fragment) (**D**). The origin of replication from the episome (855 bp fragment) and a genomic control fragment from Phatr2_52110 (188 bp) were co-amplified as a control of the samples (**E**). Lanes marked L contains GeneRuler 1 kb Plus DNA Ladder (Fermentas), WT is wild type *P. tricornutum*, PL is pPtPuc3m-Cas9_sgRNA plasmid and NTC (non-template control) is water. Numbers above lanes correspond to colony numbers indicated on the agar plates.

Delivery of the pPtPuc3m diaCas9_sgRNA episome vector to diatom cells via bacterial conjugation enables the creation of non-transgenic lines since the vector or fragments of the vector does not get integrated into the genome^13^. Previous work with diatom episomal vectors has shown that these vectors are lost in the majority of the diatom cells after growth in the absence of selection pressure^9,13^. Non-transgenic lines no longer containing the CRISPR-Cas9 episomal vector originating from single cells with confirmed bi-allelic mutations, could also be easily isolated in our study. After 16 days of growth in non-selective liquid media (cell division/day ≈1.5), single cells were transferred to non-selective agar plates. Colonies (n = 25) originating from single cells were randomly picked from the non-selective agar plates and patched onto new agar plates with or without antibiotics. A schematic overview of the experimental process for generation of non-transgenic mutant lines is presented in Supplementary Fig. 3. Following this procedure, 40–50% of the mutant colonies were found to be unable to survive on selective media, indicating loss of the pPtPuc3m diaCas9_sgRNA episome containing the *Sh Ble* selection marker (Fig. 5A,B). A segregation efficiency of 97% for the episome is identical to that reported by Karas and co-workers for the p0521s episome from which pPtPuc3 was derived^13^. The loss of the episome was further confirmed by the inability to amplify a Cas9 DNA fragment from these cells by PCR (Fig. 5C). Results from a similar experiment recently published by Slattery and coworkers^9^ indicate that a much shorter period of growth (1 week (9 generations)) in the non-selective medium than used in our study, could be sufficient to promote loss of the episome. Eight of the episome free and episome containing cell lines were re-checked after 10 months in liquid culture for presence or absence of episomes. The bacterial ori region was included as an additional episome marker and a genomic locus, Phatr2_52110 was added as a positive control. This shows that the episome is stably maintained in the cells when grown in presence of selection antibiotic (Fig. 5D,E).

## Conclusion

Bacterial conjugation-based CRISPR/Cas9 genome editing requires no expensive equipment, ensures high numbers of transformed colonies, and strongly reduces the risk of integration of vector fragments into the diatom genome compared to biolistic method. The somewhat higher mutation efficiency observed when the components necessary for CRISPR/Cas9 induced editing are expressed from stably integrated Cas9 and sgRNA genes compared to when these genes are located at an episomal vector, does not outweigh the disadvantages connected to the continuous presence and expression of the Cas9 nuclease. Elimination of the episomal CRISPR/Cas9 vector will ensure the preservation of stable knock out lines, thus limiting the chance of re-editing and off-target mutations to accumulate over time. The removal of vectors integrated into the genome through biolistic transformation is infeasible. Also, the possibility to isolate diatom CRISPR/Cas9 mutants with no transgenic DNA can be a huge benefit if the mutant strains are intended for industrial applications. CRISPR/Cas9-edited non-transgenic organisms with only small indel mutations might not be legally viewed as GMOs depending on the individual countries gene technology legislation^28,29^.

## Methods

### Strains and growth conditions

*P*. *tricornutum* cells (CCMP2561) were grown axenically and maintained as described in^7,30^. Briefly, cells were grown in f/2 medium^31^ and maintained at 22 °C under a photoperiod of 16 h light:8 h dark.

### Construction of the pPtPuc3m diaCas9_sgRNA vector for bacterial conjugation

The diaCas9-sgRNA cassette from the pKS diaCas9_sgRNA vector^7^ was inserted into a modified version of the nuclear episomal vector pPtPuc3^13^. The pPtPuc3 was purchased from Addgene (#62863) and subjected to site-directed mutagenesis to remove a single BsaI restriction site using the QuikChange II Site-Directed Mutagenesis Kit (Agilent) according to manufacturer guidelines. Mutagenic primers used were:

Puc3rBsaI.F (5′-GCAATGATACCGCGATACCCACGCTCACCG-3′) and

Puc3rBsaI.R (5′-CGGTGAGCGTGGGTATCGCGGTATCATTGC-3′). The introduced single base pair mutation is underlined in the primer sequences. The diaCas9-sgRNA cassette was assembled into the modified pPtPuc3 vector (pPtPuc3m) using the NEBuilder HiFi DNA assembly Master Mix (NEB). The diaCas9_sgRNA fragment was generated by PCR with the pKS diaCas9_sgRNA vector as template, a Q5 High-Fidelity DNA polymerase (NEB) and primers containing 5′ non-priming overlap sequences homologous to the pPtPuc3m vector sequence. Primers used in the PCR reaction to enable subsequent HiFi DNA assembly were GibLHCF2Cas.F (5′-GAGTCTGGACTTGACCTGAGCTCAATCTCGCCTATTCATGG-3′) and GibLHCF2Cas.R (5′-CGCCAAGCTTGCATGAAGCTTGCAGAAAAGTTCGTCG-3′). Overlapping sequences are underlined. Primers were designed using SnapGene software (version 3.1.4) following the recommendations in the NEBuilder HiFi DNA assembly protocol. To enable insertion of the diaCas-sgRNA fragment into the pPtPuc3m vector at a site immediately following the 3′ end of the CEN6-ARSH4-HIS3 region, the pPtPuc3m was linearized with XbaI and SphI-HF (NEB) restriction enzymes. The linearized vector and the diaCas-sgRNA fragment were assembled in a HiFi DNA assembly reaction following the manufacturer’s instructions using a vector: insert ratio of 1:2. To confirm that no mutations had been introduced due to PCR errors during amplification of the diaCas9_sgRNA fragment, the entire insert and the junction area between the insert and vector were sequenced after assembly. The vector map and sequence for the pPtPuc3m diaCas9_sgRNA is presented in Supplementary Figs 1 and 4, respectively. Plasmid map was generated using Snap Gene. The pPtPuc3m diaCas9_sgRNA plasmid is available through AddGene (ID #: 109219).

### Target gene and PAM target site selection

A Myb transcription factor (XM_002181623; named Pt_Myb1R_SHAQKYF5 in^12^ were selected for CRISPR/Cas9 gene editing using two different vectors with identical diaCas9_sgRNA cassettes, one for delivery by biolistic transformation (pKS diaCas9_sgRNA^7^) and one for bacterial conjugation (pPtPuc3m diaCas9_sgRNA). Two different PAM-target sites (Myb PAM1 and Myb PAM2) with low homology to other genomic loci were selected as described previously using an in-house PAM motif search tool^7^ to target the coding sequence of the Myb gene. Myb PAM1 was designed to introduce mutations before the single Myb binding domain (Myb1R) while Myb PAM2 was designed to induce mutations downstream of the Myb binding domain. Small adapters for the targets of interest were inserted into the sgRNA of both vectors as described in the protocol for CRISPR/Cas9 gene editing in *P*. *tricornutum*^32^.

### Introduction of CRISPR/Cas9 plasmids into *P*. *tricornutum*

#### Transformation by biolistic bombardment

*P*. *tricornutum* cells were co-transformed with the pKS diaCas9_sgRNA vector (2.5 μg) and the pAF6 vector (2.5 μg) containing a selection marker conferring resistance to zeocin by biolistic transformation as described in the *P*. *tricornutum* CRISPR/Cas9 gene editing protocol^32^. Transformed colonies appeared on selection plates after 3–6 weeks of incubation.

#### Transformation by bacterial conjugation

The pPtPuc3m diaCas9_sgRNA plasmid was delivered to *P*. *tricornutum* cells via conjugation from *Escherichia coli* DH10β cells as described by Karas and coworkers^13^. *E*.*coli* DH10β cells with mobilisation helper plasmid pTA-Mob, containing all genes necessary for the conjugative transfer of *oriT*-containing plasmids^33^, was a kind gift from the late Professor Svein Valla at NTNU. *E*. *coli* pTA-Mob competent cells (100 μl) were transformed with 200 ng of the pPtPuc3m diaCas9_sgRNA plasmid and used for conjugative delivery of the CRISPR-Cas9 plasmid to the diatoms cells. *P*. *tricornutum* cells were harvested by centrifugation (4500 rpm, 10 min) and the concentration was adjusted to 1.0 × 10^8^ cells ml^−1^ . 250 µl of the cell suspension was plated on 50% SW f/2-Si, 1% (w/v) agar plates. The plates were incubated for four days at 22 °C (16 h light and 8 h dark). Cells were scraped off from the plate using 500 µl f/2 media and the concentration was adjusted to 5.0 × 10^8^ cells ml^−1^. *E*. *coli* cells (DH10β) containing pTA-MOB and CRISPR-Cas9 plasmid (pPtPuc3m diaCas9_sgRNA) were grown in 50 ml LB media containing 50 μg/ml kanamycin and 10 μg/ml gentamicin (37 °C, 150 rpm shaking) until optical density (OD_600_) reaches 0.8–1.0. Cells were spun down for 10 min at 3000 g and resuspended in 500 µl SOC media. 200 µl of both *P*. *tricornutum* and *E*. *coli* cells were mixed together by pipetting in 1.5 ml Eppendorf tubes. Mixed cultures were plated on 50% SW f/2-Si, 5% LB, 1% (w/v) agar plates without zeocin and incubated in dark at 30 °C for 90 min. Plates was transferred to the growth room and incubated for two days at 22 °C (16 h light and 8 h dark). After 2 days the cells were transferred to 50% SW f/2-Si, 1% (w/v) agar plates containing 100 μg/ml zeocin. The plates were incubated at 22 °C (16 h light and 8 h dark). Colonies appeared on the selection plates after 10–14 days. Occasionally non or few transformants have been observed, probably as a result of plasmid instability due to the large size of the vector. Plasmid integrity should therefore be checked before *P*. *tricornutum* transformation. The isolated pPtPuc3m diaCas9_sgRNA plasmid was digested with HindIII (NEB). An intact plasmid will give 6 fragments, where 5 of the fragments can be visualized on an agarose gel (Fragment 1: 5874 bp; 2: 3161; 3: 2405 bp; 4: 1178 bp; 5: 190 bp; 6: 11 bp (off the gel)). Once the plasmid is transformed into *P*. *tricornutum* it will replicate as an episome and remain stable if propagated in zeocin containing growth media (Supplementary Fig. 5).

#### Mutant screening

Transformants were screened for targeted DNA mutations by performing high-resolution melting (HRM) based PCR analyses of the regions spanning the Myb PAM1 and PAM2 target areas as previously described^7,32^. In brief, cell lysates were generated from primary colonies, and ~500 bp PCR products flanking the target regions were amplified using primer pairs listed in Supplementary Table 3. Resulting PCR products were analyzed by 1% agarose gel electrophoresis for verification of successful PCR amplification and detection of larger indels. Highly diluted (1:4 × 10^6^) PCR products were used as template in HRM analysis for detection of small indels located in the target regions. HRM primers for amplification of short (~100 bp) PCR products are listed in Supplementary Table 2. PCR products from colonies where the HRM analyses indicated mutations in the cells, were cloned into pCR™ 4-TOPO® TA vectors (Invitrogen) to enable identification of the types of indels present in the primary mutant colonies. Plasmid DNA was isolated using E.Z.N.A.® Plasmid Mini Kit (Omega) from 6–8 randomly selected bacterial colonies resulting from transformation with TOPO vectors containing ~500 bp PCR product insert from each of the mutant diatom colonies. The inserts were sequenced by Sanger sequencing using the universal vector specific primer M13 rev (−29). Polymorphisms present in the Myb gene (Supplementary Table 1) were used to differentiate alleles for MYB1R_SHAQKYF5 gene.

### Re-plating and re-screening of colonies transformed by bacterial conjugation

Transformed primary colonies obtained by bacterial conjugation that had been screened for mutations one month after transformation were maintained on ½ f/2, 1% (w/v) agar plates containing 100 μg/ml zeocin for three months. Colonies were transferred to new plates once per month. After three months, these colonies were re-examined for targeted DNA mutation by HRM and sequencing.

### Isolation of lines with biallelic MYB mutations

Primary mutant colonies/cultures might contain both wild type and cells with a variety of different indels in the target region. Highly diluted cell cultures (approx. 300 cells/ml) from all primary mutant clones were spread onto 50% SW f/2, 1% agar plates containing 100 μg/ml zeocin to isolate single cells. Colonies originating from single cells were screened as described previously^7,32^ to identify cell lines with biallelic mutations at the Myb PAM1 and PAM2 target sites. In short, the target region was PCR amplified and direct sequenced and lines with no sign of WT alleles were selected. Target regions from candidate lines were Topo-cloned and sequenced to verify biallelic indels. Polymorphisms in the Myb gene (Supplementary Table 1) were used to distinguish between the alleles, and to verify that both alleles had been amplified and sequenced.

### Quantitative real time PCR (qRT-PCR) for determination of Cas9 gene expression level and determination of relative content of Cas9 DNA from genomic DNA isolated from transformed *P*. *tricornutum* lines

Around 2 × 10^7^ cells were collected by filtration (DVPP, 0.65 µm, Merck Millipore) from 50 ml of culture originating from single cells with biallelic mutations. Cells from filter papers were re-suspended in 1 ml of f/2 media and centrifuged for 1 min at 13000 rpm. Total RNA and genomic DNA was extracted from three mutant lines generated by each of the two CRISPR-Cas9 vectors and transformation methods. Total RNA was isolated using Spectrum Plant Total RNA Kit plant (Sigma-Aldrich) while genomic DNA was isolated using DNeasy power plant pro kit (Qiagen). DNA and RNA quantification was performed using a NanoDrop ND-1000 Spectrophotometer (NanoDrop technologies). Complementary DNA (cDNA) was synthesized from 800 ng of total RNA using QuantiTect Reverse Transcription Kit (Qiagen) following the manufacturer’s instructions. qPCR from genomic DNA was done using 5 ng of isolated genomic DNA.

Quantitative RT-PCR was performed using LightCycler 480 SYBR green I Master kit (Roche) and a LightCycler 96 instrument (Roche). PCR efficiencies were calculated for each sample using LinReg PCR software^34,35^. The Phatr2_28684 and Phatr2_24186^36^ genes were used as internal reference genes for normalizing the relative expression of Cas9 and the relative content of Cas9 DNA. Calculations of the relative Cas9 expression and Cas9 DNA content were performed using the qRT-PCR analysis software qbase+ version 2.6.1 (Biogazelle). The mean of the individual biological replicates (n = 3) was calculated and used to determine the ratio of Cas9 expression. Relative expression of Cas9 was normalized to the cell lines displaying the lowest Cas9 expression. Similar, the relative amount of Cas9 DNA was normalized to the cell lines showing lowest Cas9 DNA content.

### Episome rescue of pPtPuc3m diaCas9_sgRNA from *P*. *tricornutum*

To analyze the episome stability and integrity when cells were maintained in f/2 liquid culture with zeocin for ten months, total genomic DNA was isolated from three mutant cell lines generated using bacterial conjugation as described above. 200 ng of genomic DNA containing pPtPuc3m diaCas9_sgRNA was used to transform *E*. *coli* DH5α competent cell using heat shock^37^. The cells were transferred to 500 μl LB media and incubated at 37 °C with shaking for 1 hr. 500 μl  of the cells were transferred to kanamycin plates (50 μg/ml ) and incubated overnight at 37 °C. Between 7–40 colonies appeared on selection plates. Five random colonies were selected for plasmid isolation. Plasmids were isolated from 5 ml overnight grown culture using E.Z.N.A.® Plasmid DNA Mini Kit I following manufacturer instructions. 150–200 ng of isolated plasmid was linearized using XbaI restriction (NEB) enzyme and analyzed on 1% agarose gel. Plasmid and genomic DNA concentrations were determined with NanoDrop (ND 1000, NanoDrop technologies).

### Generation of transgene-free mutant lines

Cultures originating from single cells with biallelic mutations at the two Myb target sites (PAM1–2) containing the episomal pPtPuc3m diaCas9_sgRNA plasmid were grown for two weeks in f/2 liquid medium without antibiotics. The cell cultures were diluted to 50000 cells/ml after the first week of growth. After the second week of growth with no selection pressure, the cell cultures were diluted to approx. 300 cells/ml before being spread onto 50% SW f/2, 1% agar plates allowing growth as individual colonies for three weeks. 25 randomly picked colonies were patched onto a new non-selective agar plate in a spatial pattern specified by grid markings. After five days of growth, all 25 colonies were re-patched on 50% f/2, 1% agar plates with and without zeocin (100 μg/ml) to identify colonies that had lost the CRISPR/Cas9 episomal vector. Verification of episome free colonies was performed by PCR using Cas9-specific primers (Supplementary Table 2) and cell lysates from colonies that only survived on non-selection plates as a template. PCR reactions using cell lysates from colonies still able to grow on selective agar plates were set up as positive controls. A schematic overview of the experimental design for generating non-transgenic lines is presented in Supplementary Fig. 3.

Two episome negative and two episome containing colonies from Myb PAM1 and Myb PAM2 lines were selected and maintained for about 10 months in f/2 liquid media (f/2 media without zeocin for episome free lines and with zeocin for episome positive lines). *P*. *tricornutum* cell lysates were generated as described above from all cell lines. Episome free and episome containing lines were verified by amplifying PCR fragments from Cas9 and Ori (Origin of replication) present in the episome, while the Phatr2_52110 gene was used as positive control of the genomic DNA. *P*. *tricornutum* wild type, pure pPtPuc3m diaCas9_sgRNA plasmid and NTC were used as controls. All the primers used are presented in Supplementary Table 2.

### Calculation of segregation efficiency for pPtPuc3m diaCas9_sgRNA

Segregation efficiency (S_eff_) was calculated using the following equation^13^:

$${{\rm{S}}}_{{\rm{eff}}}={({{\rm{P}}}_{{\rm{zeocin}}}/100)}^{1/{\rm{n}}}$$

P_zeocin_ is the percentage of zeocin resistant colonies remaining after cultivation of *P*. *tricornutum* under no antibiotic selection (without zeocin) and n is the number of cell divisions estimated to have occurred in this period. In total 50 colonies were tested, 22 remained zeocin resistant and retained the episome after cultivation without zeocin selection, P_zeocin_ = 44. *P*. *tricornutum* algae were cultivated in non-selective liquid media for 16 days with ≈1.5 cell division/day and allowed to grow as individual colonies for three weeks on non-selective agar plates for 21 days ≈ 1 cell division/day. However, the re-streaking of the cells on zeocin plates indicates that the episomes are primarily lost during growth in liquid culture without selection as there is no “patchy” growth of zeocin resistant algae from re-streaked colonies. The 16 days growth on non-selective media is therefore used to estimate n. With n = 24 this gives a S_eff_ = 0.97.

## Electronic supplementary material

Supplementary Information
