# Supplementary material for: Transgene-free genome editing in marine algae by bacterial conjugation – comparison with biolistic CRISPR/Cas9 transformation
Source: Sci Rep. 2018 Sep 26;8:14401. doi: 10.1038/s41598-018-32342-0 (PMC6158232; doi:10.1038/s41598-018-32342-0)
Supplement: Supplementary file 1 — Supplementary Information [file 41598_2018_32342_MOESM1_ESM.pdf]

Supplementary information.

Transgene-free genome editing in marine algae by bacterial conjugation – comparison with biolistic CRISPR/Cas9 transformation.

Sharma AK[1], Nymark M[1], Sparstad T[1], Bones AM[1], Winge P[1].

1. Cell, Molecular biology and Genomics Group, Department of Biology, Norwegian University of Science and Technology, N-7491 Trondheim, Norway.

Supplementary Figure 1. Schematic illustration of the pPtPuc3m diaCas9_sgRNA plasmid. Important elements of the episomal plasmid pPtpuc3m_diaCas9_sgRNA: diaCas9 (brown) under regulation of LHCF2 promoter and LHCF1 terminator (grey), sgRNA (red) regulated by U6 promoter (yellow), target region (green), zeocin resistance cassette (black), origin of transfer (oriT; pink), CEN/ARS (dark grey).

Supplementary Figure 2. **Overview of CRISPR-induced targeted mutations in the Myb1R_SHAQKYF5 gene identified in primary colonies.** Different types of mutations at the targets sites Myb PAM1 and Myb PAM2 present in primary colonies by using either pKS_diaCas9_sgRNA for biolistic bombardment or pPtpuc3m_diaCas9_sgRNA for bacterial conjugation. C is used as an abbreviation for colony number. The target sequence is shown in green with PAM sequence in brown font. Indels are shown in red font.

Supplementary Figure 3: Experimental design for generating non-transgenic mutant lines. Mutant lines originating from single cells were grown in the absence of antibiotics for 16 days during which they were 100-fold diluted after 7 days. After 16 days, cells were 1000-fold diluted, and 200 µl from the cultures were plated to obtain single colonies on non-selection plate. Colonies could be picked after three weeks. Cells from 25 randomly chosen colonies were patched onto a new non-selection plate in a grid pattern. After five days randomly selected colonies were re-patched on non-selection plate and selection plate. Verification of non-transgenic cells was done by PCR.

Supplementary Figure 4. Sequence for pPtPuc3m_diaCas9_sgRNA from 5' end. oriT. Colour coding used: BleoR, CEN/ARS, LHCF2 Promoter, diaCas9, LHCF1 terminator, **U6promoter,** sgRNA, U6 3' prime region, kanR

Supplementary figure 5: Plasmid rescue of pPtPuc3m_diaCas9_sgRNA episome from transformed *P. tricornutum* lines. Plasmid rescue was performed using genomic DNA from pure mutant lines containing pPtPuc3m_diaCas9_sgRNA episome and used to transform *E. coli* Dhα. 150-200ng of isolated plasmid DNA was linearized using XbaI restriction enzyme and separated by agarose gel electrophoresis. Lanes marked with L indicate 1-kb plus ladder (Fermentas) and with PL indicate pPtPuc3m_diaCas9_sgRNA plasmid as control. CON with number indicate colonies generated using bacterial conjugation and A-E represent 5 plasmids isolated from 5 random *E. coli*.

Supplementary Table 1: List of polymorphisms used to differentiate alleles for the MYB1R_SHAQKYF5 gene. Pt_Myb (XM_002181623) sequence is used as a reference gene, and the numbers represent the position of the nucleotide in the reference gene.

Supplementary Table 2: List of primers used in the experiments.

Supplementary Figure 1

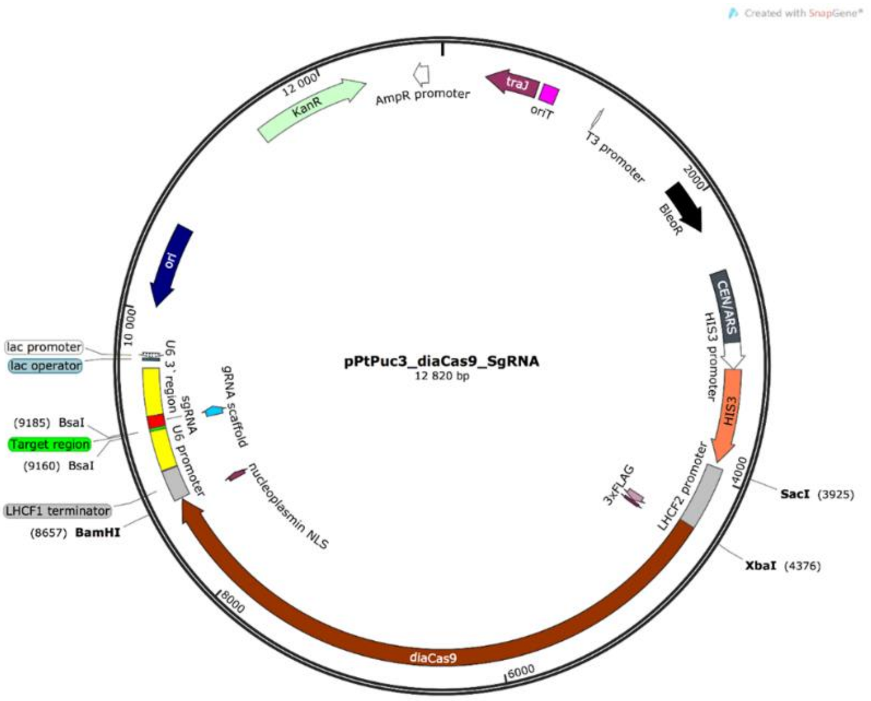

Supplementary Figure 2

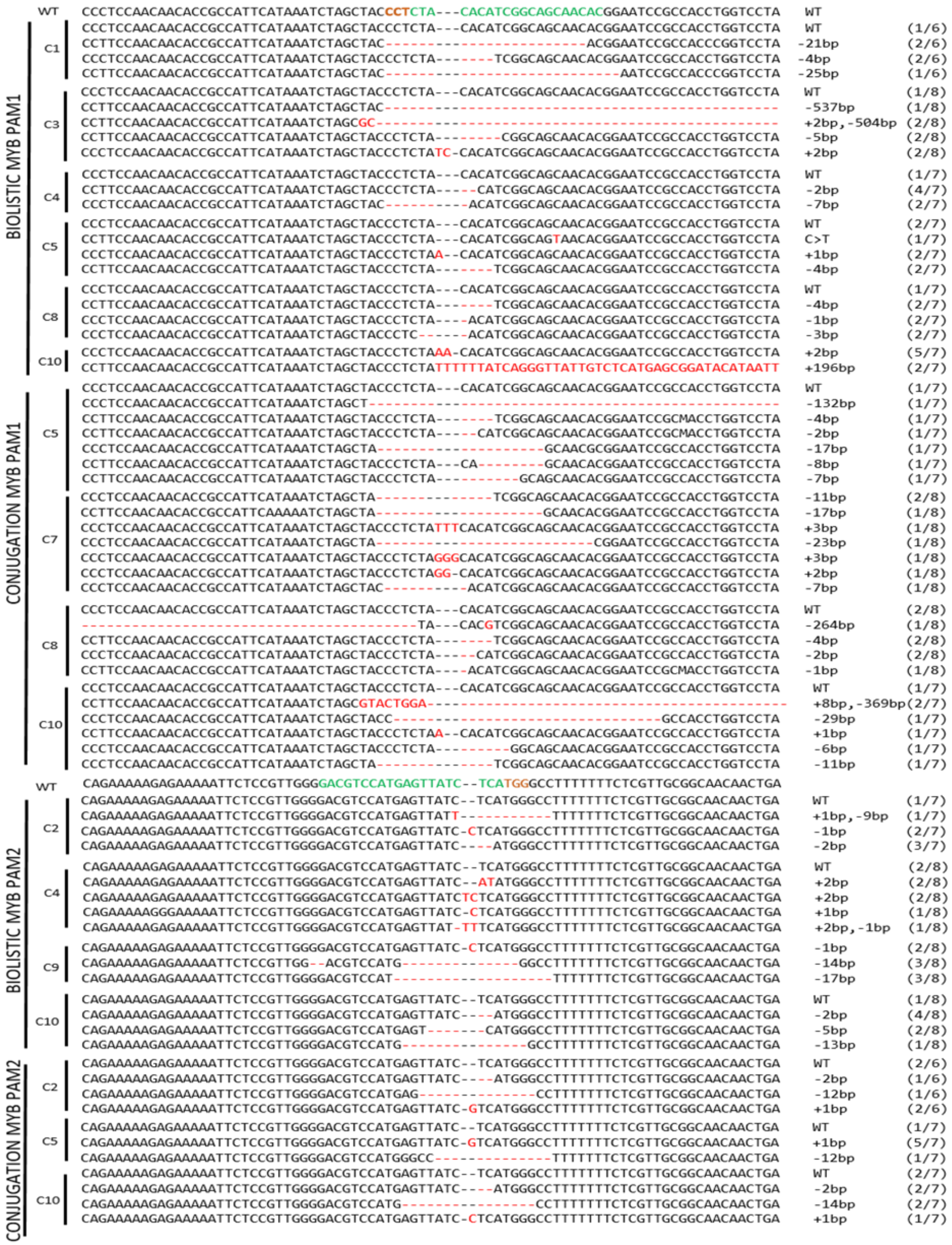

Supplementary Figure 3

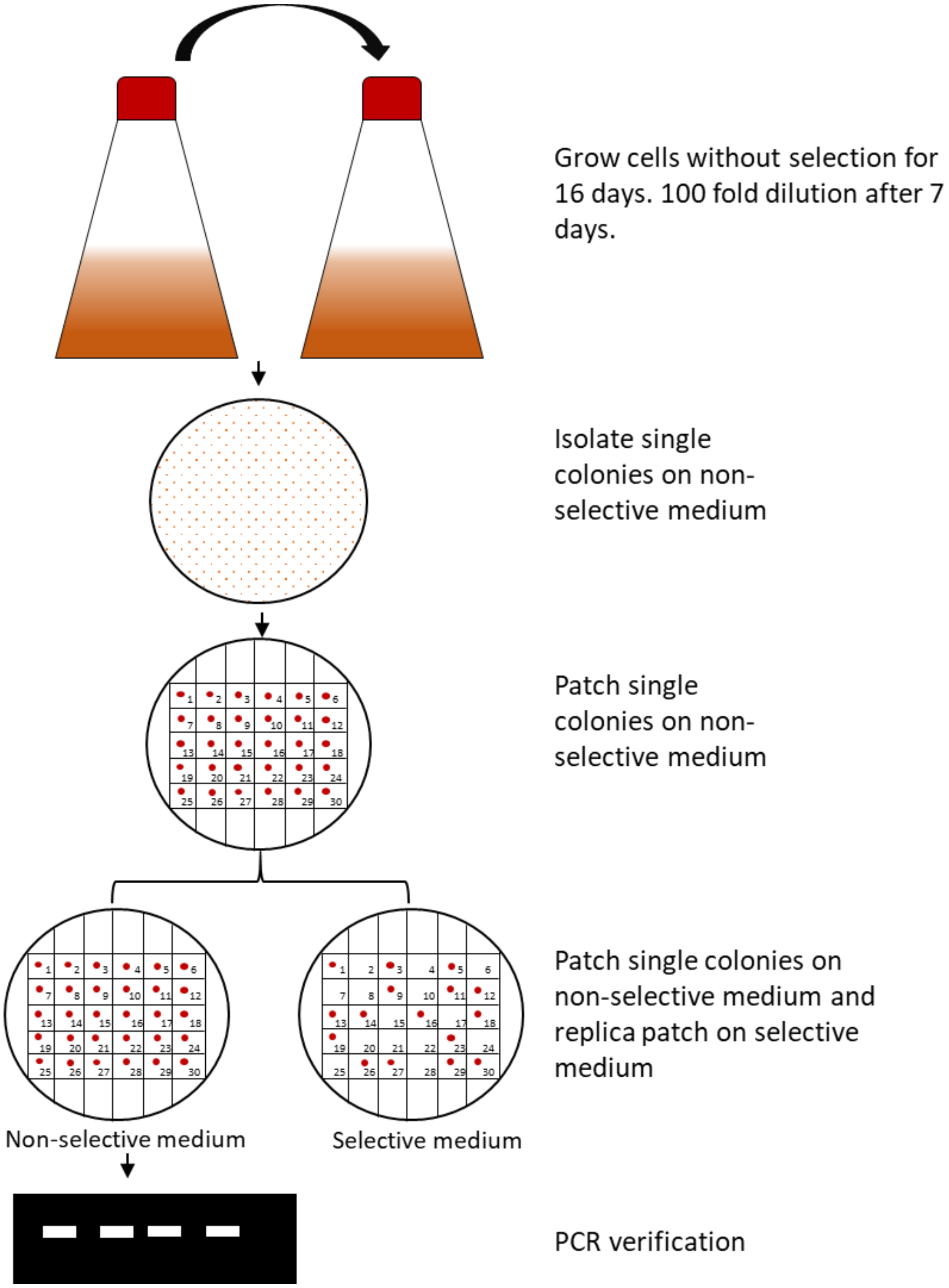

Supplementary Figure 4

TCGCGCGTTTCGGTGATGACGGTGAAAACCTCTGACACATGCAGCTCCCGGAGACGGTCACAGCTTGTCT
GTAAGCGGATGCCGGGAGCAGACAAGCCCGTCAGGGCGCGTCAGCGGGTGTTGGCGGGTGTCGGGGCTGG
CTTAACTATGCGGCATCAGAGCAGATTGTACTGAGAGTGCACCAGATCGTCTTGCCTTGCTCGTCGGTGA
TGTACTTACAGCTCGAAGTGCCTCTTCTTGATGGAGCGCATGGGGACGTGCTTGGCAATCACGCGCACCC
CCCGGCCGTTTTAGCGGCTAAAAAAGTCATGGCTCTGCCCTCGGGCGGACCACGCCCATCATGACCTTGC
CAAGCTCGTCCTGCTTCTCTTCGATCTTCGCCAGCAGGGCGAGGATCGTGGCATCACCGAACCGCGCCGT
GCGCGGGTCGTCGGTGAGCCAGAGTTTCAGCAGGCCGCCCAGGCGGCCCAGGTCGCCATTGATGCGGGCC
AGCTCGCGGACGTGCTCATAGTCCACGACGCCCGTGATTTTGTAGCCCTGGCCGACGGCCAGCAGGTAGG
CCGACAGGCTCATGCCGGCCGCCGCCTTTTCCTCAATCGCTCTTCGTTCGTCTGGAAGGCAGTACAC
CTTGATAGGTGGGCTGCCCTTCCTGGTTGGCTTGGTTTCATCAGCCATCCGCTTGCCCTCATCTGTTACG
CCGGCGGTAG**CCGGCCAGCCTCGCAGAGCAGGATTCCCGTTGAGCACCGCCAGGTGCGAATAAGGGACAG**
TGAAGAAGGAACACCCGCTCGCGGGTGGGCCTACTTCACCTATCCTGCCCGGCTGACGCCGTTGGATACA
CCAAGGAAAGTCTACACGAACCCTTTGGCAAAATCCTGTATATCGTGCGAAAAAGGATGGATATACCGAA
AAAATCGCTATAATGACCCCGAAGCAGGGTTATGCAGCGGAAGATGGCCATTCGCCATTCAGGCTGCGCA
ACTGTTGGGAAGGGCGATCGGTGCGGGCCTCTTCGCTATTACGCCAGCTGGCGAAAGGGGGATGTGCTGC
AAGGCGATTAAGTTGGGTAACGCCAGGGTTTTCCCAGTCACGACGTTGTAAAACGACGGCCAGTGACCAT
GATTACGCCAAGCTCGAAATTAACCCTCACTAAAGGGAACAAAAGCTGGTACCTAACAGGATTAGTGCAA
TTCGAGTTGAATCACTGGGAAAAACATTGTCTTCTTTTTTATATTATCATTTGCATTAGTGCTGCAGTCG
TAGATACTTGTTGGTTGAAAGACATCAGCTGGGAGGGACTGGACTAGCGTTTGGTAAGGAGACATACCTG
TTAACGTTGGTTGCAAAATTCCATTTCGCGATTTATGTTATCTGTAAATCCTGATTTGTCTGGAATTCTT
GATACTTCCGTTTTTTTAGAGGCCAATGATTAGCATCGGCGATTCTCAAAATAGCATTTTCGACATGCGG
TGCTGATTTCATAAACATAGACAACGCTTTTACATGTAAAAGTAACTTGCGGACTTGGAACAGTGCTCTG
TTTTTGGTGTGAACGTAACTCAGCAATATTTCTGTGCTAGCAAGGTTTTTTATGATCGACCGAAGATCTC
AAAACTCCGGGTCTTTCAACTGTCTGACTAGACCATGTTCGTAACGTCGGACAGCAGCTTTCGTTGTACT
GGTAGAATTTCTACGTGCGAAGCACGTGTAGGCAGGTTGAACGACGATCCCTGCCGATGGATGGATTGGC
ACGCGGCGGAACGCTTTCGTGATCTACACCACCTGGATCTTCACATATCTTCGAAATCGAAAAATTAACC
AAGTCGACGGTATCGATAATATTCTAGCTGAGGGTACCCATGGCCAAGTTGACCAGTGCCGTTCCGGTGC
TCACCGCGCGCGACGTCGCCGGAGCGGTCGAGTTCTGGACCGACCGGCTCGGGTTCTCCCGGGACTTCGT
GGAGGACGACTTCGCCGGTGTGGTCCGGGACGACGTGACCCTGTTCATCAGCGCGGTCCAGGACCAGGTG
GTGCCGGACAACACCCTGGCCTGGGTGTGGGTGCGCGGCCTGGACGAGCTGTACGCCGAGTGGTCGGAGG
TCGTGTCCACGAACTTCCGGGACGCCTCCGGGCCGGCCATGACCGAGATCGGCGAGCAGCCGTGGGGGCG
GGAGTTCGCCCTGCGCGACCCGGCCGGCAACTGCGTGCACTTCGTGGCCGAGGAGCAGGACTGACCGACG
CCGACCAACACCGCCGGTCCGACGCGGCCCGACGGGTCCGAGGCCTCGGAGATCTGGGCCCATGCGGCCG
CAACAACTACCTCGACTTTGGCTGGGACACTTTCAGTGAGGACAAGAAGCTTCAGAAGCGTGCTATCGAA
CTCAACCAGGGACGTGCGGCACAAATGGGCATCCTTGCTCTCATGGTGCACGAACAGTTGGGAGTCTCTA
TCCTTCCTTAAAAATTTAATTTTCATTAGTTGCAGTCACTCCGCTTTGGTTTCACAGTCAGGAATAACAC
TAGCTCGTCTTCAGCGAGCATCACGTGCTATAAAAATAATTATAATTTAAATTTTTTAATATAAATATAT
AAATTAAAAATAGAAAGTAAAAAAAGAAATTAAAGAAAAAATAGTTTTTGTTTTCCGAAGATGTAAAAGA
CTCTAGGGGGATCGCCAACAAATACTACCTTTTACCTTGCTCTTCCTGCTCTCAGGTATTAATGCCGAAT
TGTTTCATCTTGTCTGTGTAGAAGACCACACACGAAAATCCTGTGATTTTACATTTTACTTATCGTTAAT
CGAATGTATATCTATTTAATCTGCTTTTCTTGTCTAATAAATATATATGTAAAGTACGCTTTTTGTTGAA
ATTTTTTAAACCTTTGTTTATTTTTTTTTCTTCATTCCGTAACTCTTCTACCTTCTTTATTTACTTTCTA
AAATCCAAATACAAAACATAAAAATAAATAAACACAGAGTAAATTCCCAAATTATTCCATCATTAAAAGA
TACGAGGCGCGTGTAAGTTACAGGCAAGCGATCCTAGTACACTCTATATTTTTTTATGCCTCGGTAATGA
TTTTCATTTTTTTTTTCCACCTAGCGGATGACTCTTTTTTTTTCTTAGCGATTGGCATTATCACATAATG
AATTATACATTATATAAAGTAATGTGATTTCTTCGAAGAATATACTAAAAAATGAGCAGGCAAGATAAAC
GAAGGCAAAGATGACAGAGCAGAAAGCCCTAGTAAAGCGTATTACAAATGAAACCAAGATTCAGATTGCG

ATCTCTTTAAAGGGTGGTCCCCTAGCGATAGAGCACTCGATCTTCCCAGAAAAAGAGGCAGAAGCAGTAG
CAGAACAGGCCACACAATCGCAAGTGATTAACGTCCACACAGGTATAGGGTTTCTGGACCATATGATACA
TGCTCTGGCCAAGCATTCCGGCTGGTCGCTAATCGTTGAGTGCATTGGTGACTTACACATAGACGACCAT
CACACCACTGAAGACTGCGGGATTGCTCTCGGTCAAGCTTTTAAAGAGGCCCTAGGGGCCGTGCGTGGAG
TAAAAAGGTTTGGATCAGGATTTGCGCCTTTGGATGAGGCACTTTCCAGAGCGGTGGTAGATCTTTCGAA
CAGGCCGTACGCAGTTGTCGAACTTGGTTTGCAAAGGGAGAAAGTAGGAGATCTCTCTTGCGAGATGATC
CCGCATTTTCTTGAAAGCTTTGCAGAGGCTAGCAGAATTACCCTCCACGTTGATTGTCTGCGAGGCAAGA
ATGATCATCACCGTAGTGAGAGTGCGTTCAAGGCTCTTGCGGTTGCCATAAGAGAAGCCACCTCGCCCAA
TGGTACCAACGATGTTCCCTCCACCAAAGGTGTTCTTATGTAGTTTTACACAGGAGTCTGGACTTGACCT
GAGCT**CAATCTCGCCTATTCATGGTGTATAAAAGTTCAACATCCAAAGCTAGAACTTTTGGAAAGAGAAA**
GAATATCCGAATAGGGCACGGCGTGCCGTATTGTTGGAGTGGACTAGCAGAAAGTGAGGAAGGCACAGGA
TGAGTTTTCTCGAGACACATAGCTTCAGCGTCGTGTAGGCTAGGCAGAGGTGAGTTTTCTCGAGACATAC
CTTCAGCGTCGTCTTCACTGTCACAGTCAACTGACAGTAATCGTTGATCCGGAGAGATTCAAAATTCAAT
CTGTTTGGACCTGGATAAGACACAAGAGCGACATCCTGACATGAACGCCGTAAACAGCAAATCCTGGTTG
AACACGTATCCTTTTGGGGGCCTCCGCTACGACGCTCGCTCCAGCTGGGGCTTCCTTACTATACACAGCG
CGCATATTTCACGGTTGCCAGAAGTCAAGATGGCCTCTAGAATGGACTATAAGGACCACGACGGAGATTA
CAAGGATCATGATATTGATTACAAAGACGATGATGATAAAATGGCCCCAAAGAAGAAGCGGAAAGTCGGT
ATACACGGAGTCCCAGCAGCCGACAAGAAGTACTCGATTGGATTGGACATCGGCACCAACTCTGTTGGAT
GGGCCGTCATCACGGACGAGTACAAGGTGCCCAGCAAGAAATTCAAAGTACTGGGAAACACTGACCGCCA
TTCGATCAAGAAGAATTTGATTGGAGCTTTGCTGTTTGACAGCGGCGAAACAGCGGAAGCCACGCGGCTT
AAACGAACCGCGAGACGAAGATATACGCGACGGAAGAACCGTATCTGCTATCTACAAGAGATTTTCTCGA
ATGAGATGGCAAAAGTCGATGACTCTTTCTTTCACCGATTGGAAGAATCCTTCCTCGTTGAAGAGGATAA
GAAACATGAGCGGCACCCGATATTTGGAAACATTGTGGACGAGGTGGCTTACCATGAGAAATACCCGACT
ATTTACCACCTGCGAAAGAAATTGGTGGACTCGACGGACAAAGCGGATCTTCGCCTAATCTATTTGGCGC
TAGCACATATGATTAAGTTTCGCGGCCACTTCTTGATCGAAGGCGACTTGAATCCGGACAACAGTGATGT
TGACAAACTGTTTATTCAACTTGTACAAACTTACAACCAGCTCTTTGAAGAAAATCCCATTAACGCTTCG
GGAGTGGACGCGAAAGCCATCCTTTCTGCCCGACTCAGCAAGAGCCGACGCCTAGAAAATTTGATTGCGC
AACTTCCCGGCGAGAAAAAGAATGGACTTTTTGGAAATCTTATTGCTTTGTCGCTGGGCCTTACGCCGAA
CTTTAAGAGCAACTTCGACCTTGCGGAAGATGCCAAATTGCAACTGAGTAAAGACACATATGACGACGAC
CTCGACAATCTTTTGGCACAAATAGGAGATCAGTACGCCGACCTCTTTTTGGCGGCTAAGAACTTGTCCG
ACGCCATTCTGTTGTCGGACATCCTTCGAGTTAATACGGAGATTACTAAGGCGCCCTTGAGCGCCTCTAT
GATCAAAAGATACGACGAGCATCACCAAGACCTGACCCTCCTTAAAGCTCTCGTCCGGCAACAGCTGCCT
GAGAAGTACAAAGAGATTTTCTTTGATCAAAGCAAAAACGGATACGCGGGCTACATTGACGGGGGAGCCT
CGCAAGAAGAGTTCTACAAGTTTATTAAACCGATCCTGGAAAAGATGGACGGCACGGAGGAATTACTCGT
GAAACTTAACCGAGAGGACCTTTTGCGTAAGCAACGGACTTTCGACAACGGATCTATTCCGCACCAAATC
CATCTCGGAGAATTGCACGCAATTCTGCGCCGGCAAGAAGATTTTTATCCATTTCTCAAGGACAACCGGG
AAAAGATTGAGAAAATCTTAACCTTCCGCATACCCTACTACGTCGGCCCTTTGGCCAGGGGAAATTCGAG
ATTTGCCTGGATGACGCGAAAGAGTGAGGAAACTATTACTCCCTGGAATTTCGAAGAAGTGGTAGACAAA
GGAGCTTCCGCGCAATCCTTTATCGAGCGCATGACCAACTTCGATAAGAACCTCCCGAATGAGAAAGTCC
TACCCAAGCATTCGTTGCTCTACGAATACTTTACGGTGTATAATGAGCTGACTAAAGTCAAATACGTTAC
AGAGGGAATGCGAAAACCGGCTTTCCTCTCGGGAGAGCAGAAAAAGGCCATAGTCGATCTTCTCTTTAAA
ACGAACCGTAAAGTTACCGTGAAACAACTAAAAGAGGACTACTTCAAGAAAATCGAATGCTTTGACTCCG
TTGAAATTTCCGGCGTTGAAGATCGGTTCAATGCCTCCTTGGGAACATACCACGATCTCCTGAAAATTAT
CAAAGACAAGGACTTTCTTGACAATGAGGAAAACGAGGACATTCTTGAAGATATTGTCTTGACGCTAACA
CTTTTTGAGGATCGAGAGATGATTGAGGAACGCCTTAAAACCTATGCGCATCTGTTCGACGACAAAGTTA
TGAAGCAATTGAAGCGCCGGAGATATACGGGATGGGGAAGGTTGAGTCGGAAACTGATAAATGGCATACG
CGATAAGCAGTCCGGCAAAACAATTCTCGATTTCTTGAAGTCCGACGGATTTGCGAATCGAAACTTCATG
CAACTGATCCATGACGATTCGTTGACCTTTAAAGAGGACATTCAAAAAGCCCAAGTATCCGGACAAGGAG
ATTCTTTGCACGAGCATATTGCGAATCTTGCTGGAAGTCCGGCTATTAAAAAGGGCATTCTCCAGACAGT

AAAGGTCGTGGATGAACTCGTTAAAGTTATGGGCCGACACAAACCCGAGAACATAGTCATCGAAATGGCG
AGAGAGAACCAGACCACCCAAAAGGGACAGAAAAATTCACGCGAGCGAATGAAGCGGATTGAAGAGGGGA
TCAAAGAGCTTGGCAGCCAAATTCTGAAAGAACACCCGGTTGAAAACACCCAATTGCAGAACGAGAAGCT
CTACCTGTACTATCTTCAAAATGGGCGTGATATGTACGTCGATCAAGAATTGGACATCAACCGGCTATCC
GATTACGATGTGGACCATATTGTTCCTCAGTCGTTTCTCAAAGACGACTCCATAGACAATAAGGTTCTAA
CGAGAAGCGACAAGAACCGCGGCAAATCAGACAACGTCCCCTCCGAGGAAGTAGTTAAGAAAATGAAGAA
TTACTGGCGCCAATTGCTGAACGCTAAGCTCATTACCCAAAGAAAGTTTGACAATTTGACGAAAGCCGAG
CGAGGCGGGCTCTCGGAACTGGATAAGGCCGGCTTCATTAAAAGACAATTGGTGGAAACGCGACAAATCA
CAAAGCACGTCGCACAGATTCTCGACTCCCGCATGAACACTAAGTATGACGAGAATGACAAACTAATCCG
GGAAGTCAAAGTGATTACCTTGAAGTCCAAGCTGGTTTCCGATTTTCGTAAAGATTTTCAGTTTTACAAA
GTTCGCGAGATCAACAACTACCATCACGCGCACGACGCTTACTTGAACGCTGTCGTGGGAACGGCCTTGA
TTAAAAAATACCCTAAGCTGGAAAGCGAGTTCGTTTACGGCGATTACAAGGTGTATGACGTACGCAAAAT
GATCGCGAAGTCAGAGCAGGAAATTGGCAAAGCTACTGCGAAGTACTTCTTTTATAGCAACATAATGAAT
TTTTTCAAAACGGAGATTACCCTCGCTAACGGCGAGATTCGCAAGCGCCCTCTTATCGAGACAAACGGCG
AAACGGGGGAGATTGTCTGGGATAAAGGCCGCGATTTTGCGACTGTGCGGAAAGTCTTGTCGATGCCGCA
AGTGAATATAGTCAAAAAGACGGAGGTGCAAACAGGCGGCTTTTCGAAAGAATCTATCCTTCCCAAAAGG
AACTCTGATAAGTTGATTGCGCGAAAAAAGGATTGGGACCCTAAGAAGTATGGCGGCTTTGACAGCCCGA
CCGTAGCATATTCTGTCCTTGTGGTGGCCAAAGTCGAAAAGGGCAAATCCAAGAAACTGAAGAGTGTGAA
AGAATTGCTTGGGATTACGATCATGGAAAGATCATCTTTTGAGAAGAATCCCATCGACTTTCTCGAAGCT
AAAGGCTACAAAGAAGTGAAAAAGGACCTGATTATCAAGTTGCCTAAGTACTCCTTGTTTGAGCTGGAAA
ATGGCCGTAAGCGAATGCTCGCTTCTGCCGGCGAACTCCAGAAGGGAAACGAATTGGCGCTGCCCTCCAA
ATATGTTAACTTTCTTTACCTTGCCTCGCACTATGAGAAGCTCAAGGGCTCCCCCGAGGATAATGAGCAA
AAACAACTGTTTGTAGAACAGCATAAGCACTACTTGGACGAGATCATTGAGCAAATTAGCGAGTTCTCCA
AGCGAGTCATCCTTGCCGACGCTAATCTGGACAAAGTCCTGTCCGCCTACAATAAGCACCGCGATAAGCC
AATCCGAGAGCAAGCGGAGAATATTATCCACTTGTTTACGCTCACCAATCTTGGAGCCCCTGCCGCCTTT
AAGTACTTTGACACCACCATCGACCGCAAGAGGTACACCAGCACCAAAGAAGTCTTGGACGCCACCCTCA
TTCACCAATCAATCACCGGCTTGTACGAGACACGCATCGACCTGTCTCAACTCGGAGGCGACAAAAGGCC
GGCGGCCACGAAAAAGGCCGGCCAGGCAAAAAAGAAAAAGTAATCTGGATCCCTACCTCGACTTTGGCTG
GGACACTTTCAGTGAGGACAAGAAGCTCCAGAAGCGTGCTATCGAACTCAACCAGGGACGTGCGGCACAA
ATGGGCATCCTTGCTCTCATGGTGCACGAACAGTTGGGAGTCTCTATCCTTCCTTAAAAATTTAATTTTC
ATTAGTTGCAGTCACTCCGCTTTGGTTTCACAGTCAGGAATAACACTAGCTCGTCTTCACTGCAG**GTTGG**
CTCGGAAGTTGGTGTTGACGGTGAGCTGGAAATTGGTTGTCGGTCACTGCTAGCGAGAAGAAAACGGAGG
ACAGAAGGAAGTGAAACTCGGTTCGTTCTCGACAGCCTCACTGTCAATATGCTCATTTTCAATCCTTAGC
GCTTTTAATGTCGAATTGACGGTAAATTGAATAGGATCTATAATATCTACAAGGTACTTTGACACGCCAA
GTATTCATTGTTAGTCAACAATATTTTAGAGCTTTATAAGGTCAAAAAACACCTTCAAAGTCGAGGAGAC
CGAGAGAGGGTCTCAGTTTTAGAGCTAGAAATAGCAAGTTAAAATAAGGCTAGTCCGTTATCAACTTGAA
AAAGTGGCACCGAGTCGGTGCTTTTTTTTAGAACCGCTCACCCATGCTATCGTATGTCATTTACATTGAC
AAACTGCTATGAATGCGTTTGCGATAACTTCAGCTCGCTGAAGCCCTAGACATTTCGTTTGACTAAATTG
TTTTCCGGTTGCTTTACTACCGACCGGTCTGCGGGTAGCATGTCTTGGCTTTTGCGAGTTCGAGAGAATT
TATGGACAAGTGCGTTTACAGTTTGGAACACGAGTGTGAGTGAGCGGAATTGGCTATCTATAACATTCTT
ATTTCAGGAGGCTGGTGATTAGCAGAATTTTCAAAGATCTCGAATCTAAAATCGCCATGGGCTCGACGAA
CTTTTCTGCAAGCTTCATGCAAGCTTGGCGTAATCATGGTCATAGCTGTTTCCTGTGTGAAATTGTTATC
CGCTCACAATTCCACACAACATACGAGCCGGAAGCATAAAGTGTAAAGCCTGGGGTGCCTAATGAGTGAG
CTAACTCACATTAATTGCGTTGCGCTCACTGCCCGCTTTCCAGTCGGGAAACCTGTCGTGCCAGCTGCAT
TAATGAATCGGCCAACGCGCGGGGAGAGGCGGTTTGCGTATTGGGCGCTCTTCCGCTTCCTCGCTCACTG
ACTCGCTGCGCTCGGTCGTTCGGCTGCGGCGAGCGGTATCAGCTCACTCAAAGGCGGTAATACGGTTATC
CACAGAATCAGGGGATAACGCAGGAAAGAACATGTGAGCAAAAGGCCAGCAAAAGGCCAGGAACCGTAAA
AAGGCCGCGTTGCTGGCGTTTTTCCATAGGCTCCGCCCCCCTGACGAGCATCACAAAAATCGACGCTCAA
GTCAGAGGTGGCGAAACCCGACAGGACTATAAAGATACCAGGCGTTTCCCCCTGGAAGCTCCCTCGTGCG

CTCTCCTGTTCCGACCCTGCCGCTTACCGGATACCTGTCCGCCTTTCTCCCTTCGGGAAGCGTGGCGCTT
TCTCATAGCTCACGCTGTAGGTATCTCAGTTCGGTGTAGGTCGTTCGCTCCAAGCTGGGCTGTGTGCACG
AACCCCCCGTTCAGCCCGACCGCTGCGCCTTATCCGGTAACTATCGTCTTGAGTCCAACCCGGTAAGACA
CGACTTATCGCCACTGGCAGCAGCCACTGGTAACAGGATTAGCAGAGCGAGGTATGTAGGCGGTGCTACA
GAGTTCTTGAAGTGGTGGCCTAACTACGGCTACACTAGAAGAACAGTATTTGGTATCTGCGCTCTGCTGA
AGCCAGTTACCTTCGGAAAAAGAGTTGGTAGCTCTTGATCCGGCAAACAAACCACCGCTGGTAGCGGTGG
TTTTTTTGTTTGCAAGCAGCAGATTACGCGCAGAAAAAAAGGATCTCAAGAAGATCCTTTGATCTTTTCT
ACGGGGTCTGACGCTCAGTGGAACGAAAACTCACGTTAAGGGATTTTGGTCATGAGATTATCAAAAAGGA
TCTTCACCTAGATCCTTTTAAATTAAAAATGAAGTTTTAAATCAATCTAAAGTATATATGAGTAAACTTG
GTCTGACAGTTACCAATGCTTAATCAGTGAGGCACCTATCTCAGCGATCTGTCTATTTCGTTCATCCATA
GTTGCCTGACTCCCCGTCGTGTAGATAACTACGATACGGGAGGGCTTACCATCTGGCCCCAGTGCTGCAA
TGATACCGCGAtACCCACGCTCACCGGCTCCAGATTTATCAGCAATAAACCAGCCAGCCGGAAGGGCCGA
GCGCAGAAGTGGTCCTGCAACTTTATCCGCCTCCATCCAGTCTATTAATTGTTGCCGGGAAGCTAGAGTA
AGTAGTTCGCCAGTTAATAGTTTGCGCAACGTTGTTGCCATTGCTACAGGCATCGTGGTGTCACGCTCGT
CGTTTGGTATGGCTTCATTCAGCTCCGGTTCCCAACGATCAAGGCGAGTTACATGATCCCCCATGTTGTG
CAAAAAAGCGGTTAGCTCCTTCGGTCCTCCGATCGTTGTCAGAAGTAAGTTGGCCGCAGTGTTATCACTC
ATGGTTATGGCAGCACTGCATAATTCTCTTACTGTCATGCCATCCGTAAGATGCTTTTCTGTGACTGGTG
AGTCGATTTATTCAACAAAGCCACGTTGTGTCTCAAAATCTCTGATGTTACATTGCACAAGATAAAAATA
TATCATCATGAACAATAAAACTGTCTGCTTACATAAACAGTAATACAAGGGGTGTTATGAGCCATATTCA
ACGGGAAACGTCTTGCTCGAGGCCGCGATTAAATTCCAACATGGATGCTGATTTATATGGGTATAAATGG
GCTCGCGATAATGTCGGGCAATCAGGTGCGACAATCTATCGATTGTATGGGAAGCCCGATGCGCCAGAGT
TGTTTCTGAAACATGGCAAAGGTAGCGTTGCCAATGATGTTACAGATGAGATGGTCAGACTAAACTGGCT
GACGGAATTTATGCCTCTTCCGACCATCAAGCATTTTATCCGTACTCCTGATGATGCATGGTTACTCACC
ACTGCGATCCCCGGGAAAACAGCATTCCAGGTATTAGAAGAATATCCTGATTCAGGTGAAAATATTGTTG
ATGCGCTGGCAGTGTTCCTGCGCCGGTTGCATTCGATTCCTGTTTGTAATTGTCCTTTTAACAGCGATCG
CGTATTTCGTCTCGCTCAGGCGCAATCACGAATGAATAACGGTTTGGTTGATGCGAGTGATTTTGATGAC
GAGCGTAATGGCTGGCCTGTTGAACAAGTCTGGAAAGAAATGCATAAGCTTTTGCCATTCTCACCGGATT
CAGTCGTCACTCATGGTGATTTCTCACTTGATAACCTTATTTTTGACGAGGGGAAATTAATAGGTTGTAT
TGATGTTGGACGAGTCGGAATCGCAGACCGATACCAGGATCTTGCCATCCTATGGAACTGCCTCGGTGAG
TTTTCTCCTTCATTACAGAAACGGCTTTTTCAAAAATATGGTATTGATAATCCTGATATGAATAAATTGC
AGTTTCATTTGATGCTCGATGAGTTTTTCTAATCAGAATTGGTTAATTGGTTGTAACACTGGCACTCAAC
CAAGTCATTCTGAGAATAGTGTATGCGGCGACCGAGTTGCTCTTGCCCGGCGTCAATACGGGATAATACC
GCGCCACATAGCAGAACTTTAAAAGTGCTCATCATTGGAAAACGTTCTTCGGGGCGAAAACTCTCAAGGA
TCTTACCGCTGTTGAGATCCAGTTCGATGTAACCCACTCGTGCACCCAACTGATCTTCAGCATCTTTTAC
TTTCACCAGCGTTTCTGGGTGAGCAAAAACAGGAAGGCAAAATGCCGCAAAAAAGGGAATAAGGGCGACA
CGGAAATGTTGAATACTCATACTCTTCCTTTTTCAATATTATTGAAGCATTTATCAGGGTTATTGTCTCA
TGAGCGGATACATATTTGAATGTATTTAGAAAAATAAACAAATAGGGGTTCCGCGCACATTTCCCCGAAA
AGTGCCACCTGACGTCTAAGAAACCATTATTATCATGACATTAACCTATAAAAATAGGCGTATCACGAGG
CCCTTTCGTC

Supplementary Figure 5

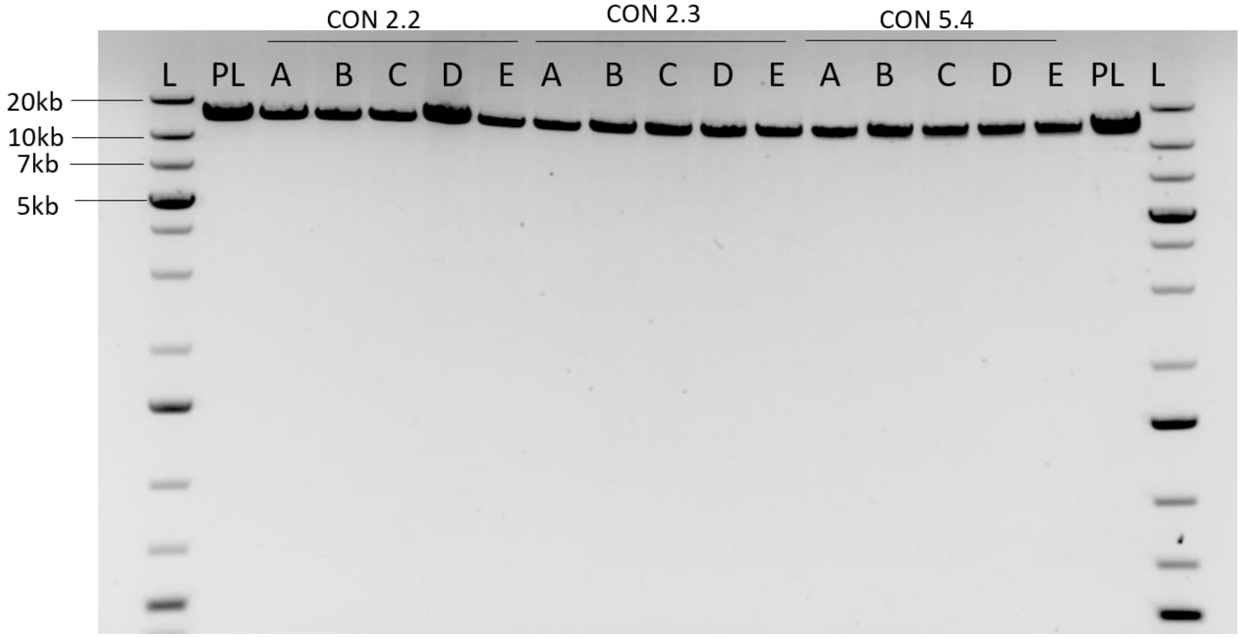

Supplementary Table 1

Nucleotide Position	Allele 1	Allele 2
112	G	A
191-196	-	GCGGCAACA (Insert)
311-312	TT	CC
330	T	C
459	T	G
477	T	C
593	G	C
600	C	G
758	G	C
1095	A	G

Supplementary Table 2: List of primers used in the experiments.

Primer Name	5' Sequence 3'	PCR product size(bp)
**Primary PCR amplification**		
Myb PAM 1 seq Fw	CCCGATGCTGCCAATCTCAGC	790
Myb PAM 1 seq Rw	GGACGGTGGCGTGCATCAAG	
Myb PAM 2 seq Fw	GCCTACGATGCGACTGTCCG	482
Myb PAM 2 seq Rw	CCGACAGCGGTGGCCGTGCTC	
**High resolution melting primers**		
HRM_Myb PAM 1_Fw	CCAACAACACCGCCATTCATAAATCTAGC	93
HRM_Myb PAM 1_Rw	GCAAATCAGTAGTCTCGTAGGACCAGG	
HRM_Myb PAM 2_Fw	GCATTGATGCTTCCGCAACTTACAG	106
HRM_Myb PAM 2_Rw	CCATCAGTTGTTGCCGCAACG	
**Verification of Construct(plasmid)**		
Myb PAM 1 Fw	CTACACATCGGCAGCAACAC	544
M13 Rev	CAGGAAACAGCTATGAC	
Myb PAM 2 Fw	GACGTCCATGAGTTATCTCA	544
M13 Rev	CAGGAAACAGCTATGAC	
**Topo sequencing**		
M13 Rev	CAGGAAACAGCTATGAC	
**Verification of marker free mutants**		
dCas9 F1	GAAGTACTCGATTGGATTGGAC	817
dCas9R1	GAGGTCGTCGTCATATGTGTCT	
OriFw	GAATCAGGGATAACGCAGG	855
OriRW	GCATTGGTAACTGTCAGACC	
Phatr2_52110F(ENTPPL)	CGTTTGTGTGCTCCGTTCTA	188
Phatr2_52110R(ENTPPL)	CCTTAAGGCATTGGAGTCACTC	
**qPCR**		
qPCRphat_28684F	TGCGCTCGACACCATTTCAA	137
qPCRphat_28684R	GCTCCTTTGAAGATTGGACG	
Phatr2_24186F	TCTCCGATACGGGCTTGGA	160
Phatr2_24186R	CAGCCGATCCGTCATAACTGC	
dCas9R4	ATATTCACTTGCGGCATCGACA	97
dCas9F5	TTATCGAGACAAACGGCGAAAC	
